# Fostering Cultures of Sustainability in a Multi-Unit Office Building: A Theory of Change

**DOI:** 10.3389/fpsyg.2021.624311

**Published:** 2021-05-10

**Authors:** Bianca Christel Dreyer, Manuel Riemer, Brittany Spadafore, Joel Marcus, Devon Fernandes, Allan Taylor, Stephanie Whitney, Sean Geobey, Aisling Dennett

**Affiliations:** ^1^Viessmann Centre for Engagement and Research in Sustainability, Waterloo, ON, Canada; ^2^Department of Psychology, Wilfrid Laurier University, Waterloo, ON, Canada; ^3^School of Administrative Studies, York University, Toronto, ON, Canada; ^4^Humber College, Toronto, ON, Canada; ^5^Sustainable Waterloo Region, Waterloo, ON, Canada; ^6^Office of Research Services, Wilfrid Laurier University, Waterloo, ON, Canada; ^7^Waterloo Institute for Social Innovation and Resilience, University of Waterloo, Waterloo, ON, Canada

**Keywords:** culture of sustainability, theory of change, behavior change, sustainability, systems thinking, culture, engagement, participation

## Abstract

Psychological approaches to fostering sustainability are heavily focused on individual behaviors and often insufficiently address the physical and social contexts individuals are embedded in. This limits the ability to create meaningful, long-lasting change, as many of day-to-day behaviors are social practices embedded in broader cultural norms and systems. This is particularly true in the work context, where organizational cultures heavily condition both the actions of individual employees and the collective actions of organizations. Thus, we argue cultures, not behaviors, must become the focus of sustainability change efforts. In this paper, we present a theory of change aimed at fostering strong organizational cultures of sustainability (COS) within a high-performance multi-tenant office building. Our theory takes a systems perspective that incorporates the social and physical aspects of the work environment, and views culture change as a co-creative exercise involving engagement of multiple stakeholders. The paper concludes with implications for practice and research.

## Introduction

“It is not only in the external physical environment, but just as much in our cultures […] that change has to take place, if we are to have a world that is sustainable for the human race in the future” (Packalén, 2010, p. 121).

There is growing recognition that significant cultural transformations are needed to successfully respond to ongoing global crises, such as the climate change crisis (Packalén, 2010). However, solutions have been primarily focused on technical innovations rather than culture shifts (Agyeman, 2005a). We were faced with this discrepancy when our team was approached in 2016 by a local environmental Non-Governmental Organization (NGO) with an opportunity to contribute to the ideation of a multi-tenant high-performance office building. Together with several partners (the leadership team), this NGO wanted to create a building that is not only carbon-neutral and regenerative, a building “that gives back” (See Riemer et al., 2021, for the story of this building), but is also commercially viable so it could be easily replicated.

High-performance buildings (HPBs), also referred to as “green” or “sustainable” buildings, can be defined as structures created with the intention of reducing resource use, emissions, and waste, while increasing occupant well-being and health (Brown et al., 2010). Yet, based on experiences of the Centre for Interactive Research in Sustainability (Fedoruk et al., 2015) and other buildings like it, there are multiple gaps between design and performance, despite the use of cutting-edge technologies and sustainable design. While there are many reasons for these performance gaps, one reason is believed to be the (in-)actions of building citizens, and more specifically building managers and organizational employees (Fedoruk et al., 2015; Coleman and Robinson, 2018). Our addition to the leadership team offered expertise related to fostering human actions that could support the performance goals of the building and realize its promise as an adaptation to the global climate crisis. We knew this required an approach that went beyond a one-off behavior change program, and instead focused on the development of building wide self-sustaining cultures^1^ of sustainability.

A scan of the literature for systemic approaches to creating and maintaining organizational- and building-level cultures of sustainability (COS) in a multi-tenant HPB, provided insufficient resources for the development of practical guidelines. This led to our decision to create a theory of change of how to co-create such cultures, building on existing work of HPBs and (organizational) change toward sustainability (e.g., Pelletier and Aitken, 2014). A theory of change “is essentially a comprehensive description and illustration of how and why a desired change is expected to happen in a particular context” (Center for Theory of Change, n.d.). A theory of change is not meant to be the same as a scientific theory with testable hypotheses as is common in psychology, but rather a theory-informed framework providing guidance to a practical approach of creating meaningful change for a specific issue. For this purpose, we engaged in “theory-knitting” (Kalmar and Sternberg, 1988) by integrating a variety of existing theories into one comprehensive applied theory of change (Riemer and Bickman, 2011). In this approach, “one integrates the best aspects of a set of given theories with one’s own ideas regarding the domain under investigation” (Kalmar and Sternberg, 1988, p.153), in our case fostering COS. While this is a useful approach for dealing with complex applied phenomena and to overcoming the limiting reductionism inherent in many psychological theories, it is not without its challenges. For example, it is crucial to ensure that the integrated theories do not rest upon incompatible basic assumptions and paradigms. It is important to note that the presented theory of change framework was primarily developed based on theoretical applications and existing literature at the time we created it, and therefore represents our expectation of what would happen once implemented.^2^ With agreement from the building citizens and leadership team, a living lab concept was incorporated into the building design and operation and served as a mechanism for both the implementation and evaluation of our approach. Thus establishing an onsite laboratory for experimentation in sustainable transformations and practical solutions for real-world problems (Heiskanen et al., 2018; Laakso, 2019). In a forthcoming paper, we will be sharing our experience and the challenges of operationalizing and implementing this theory of change. In this paper, we will first discuss the relevance of cultures of sustainability for achieving the goals of high-performance office buildings (and sustainability more broadly). This will provide the context that informed our approach. We will then offer our theory of change as a system-oriented framework informed by bottom-up engagement processes and discuss its potential challenges and their potential solutions, followed by a general conclusion.

## Cultures and Sustainability in the Built Environment

Conceptualizing our theory of change required understanding how sustainability and change has been considered and integrated in both the built environment and organizations themselves. In the last 2 decades, sustainability has become synonymous with the necessity of integrating the imperatives of environmental protection, economic development, and social justice – the so-called tri-factor of sustainability (Marcus et al., 2010). At the same time, there are growing concerns about how sustainability change efforts can facilitate integrated thinking, while they continue to apply this typology. Gibson et al. (2005, p. 94) argue that decision-makers are “struggling to understand the overall implications of separate ecological, social, and economic assessment reports that are integrated only by the staples holding the documents together.” Social justice and equity (including economic equity) are integrally part of achieving just and sustainable futures; they cannot be considered separately, and we cannot have one without the other (Rauschmayer et al., 2015; see also discussions of “just sustainabilities” literature that argues for a strong connection of social justice and environmental sustainability, e.g., Agyeman, 2005a,b, 2008). Thus, we echo others in asserting the need to ensure justice is “an essential and integral part of systemic change” (van Steenbergen and Schipper, 2017, p.8). In order to assess and “achieve” sustainability, its core elements need to be integrated. This requires changes both in the conceptualization and implementation of sustainability change efforts. Specifically, a stronger focus on social systems and the underlying cultures that shape the system structures and behavior patterns is needed. Given our context of a multi-tenant office building, we will first explore current conceptualizations of sustainability change in the built environment and among organizations. We will then discuss how the focus on cultures can address current tensions in the sustainability and (organizational) change literatures, and end with a discussion of core principles of cultures of sustainability.

### Sustainability in the Built Environment

The concept of “sustainable” buildings has further complicated an already complex concept. While some argue that buildings are inherently unsustainable, others argue what is needed is a focus on making them sustainable (Robinson and Cole, 2015). This call for action has resulted in significant innovations in technology and governance models for individual building systems; mostly focused on transitions to a low-carbon economy (Foxon, 2011). Third party certification bodies, for example, focus heavily on technology and design, such as the Leadership in Energy and Environmental Design (LEED) rating system (Azhar et al., 2011), with the intention of reducing the environmental impact of buildings through carbon reductions. Unfortunately, HPBs frequently fail to meet their expected reduction targets, a phenomenon coined the “performance gap” (Fedoruk et al., 2015; Coleman et al., 2018). Coleman et al. (2018), however, point out that limiting the performance gap to energy and other carbon reduction targets misses others related to the impact of buildings on its citizens, through indoor environmental quality and social factors, or on society at large.

A more nuanced notion of performance gaps could consider gaps between predicted vs. actual resource use (such as energy, water, and waste), measured vs. perceived indoor environmental conditions (such as temperature, air, and lighting; Fabbri and Tronchin, 2015; Phillips and Levin, 2015; Tuohy and Murphy, 2015) and expected vs. actual lived experiences (such as equity, well-being, comfort, and productivity; De Wilde, 2014; Fedoruk et al., 2015; Coleman and Robinson, 2018). These gaps can also have synergistic impacts with one another. The current trend of designing for carbon reductions, such as energy performance improvements, can contradict measures for optimal indoor environmental quality or equity and well-being for building citizens (Wargocki and Wyon, 2013; Arif et al., 2016; Baloch et al., 2020).^3^ For example, a HPB may not provide any ability to control the indoor environment (e.g., adjust temperatures), creating occupant discomfort and a narrow focus on carbon reductions of the built environment has wider implications on social and economic sustainabilities through housing affordability, fuel poverty, and health inequities (Shrubsole et al., 2019a). It is partly due to the failure to consider buildings as dynamic systems within wider contexts that make these low-carbon transitions prone to negative and unintentional consequences (Janda, 2011). Building performance and sustainability goals thus need to be expanded, as buildings are part of wider socio-economic activities and cultural practices and they play a crucial role in many aspects of people’s lives (Shrubsole et al., 2019a).

As argued elsewhere (Shove, 2010; Rauschmayer et al., 2015; Fischer and Newig, 2016; Geels, 2020), understanding and addressing the causes of (un)sustainability raises the question of whether to tackle individual or structural factors, or perhaps to find adequate ways for a combination of both. Finding this dialectic is contended to be a prerequisite of sustainability. Thus, it is imperative to understand the role of building citizens and other stakeholders (individuals) and (organizational) structures in working toward the sustainability goals of HPBs; especially in office buildings where employees often spend a third of their day (Dreyer et al., 2018). Thus, a theory of change intended to foster sustainability within this context ought to consider these complexities.

### Transitioning Organizations Toward Sustainability

In conceptualizing fostering changes, or “transitions” of (building and organizational) systems toward sustainability it is useful to consider the contributions that transition management literature has made toward understanding these processes. Transitions are understood as changes in the regime, “conglomerates of structure (physical setting), culture (prevailing perspective), and practices (rules, routines, and habits)” (Rotmans and Loorbach, 2009, p. 185). A regime change can be influenced by three interlocking dynamics: top-down (pressures of context, i.e., landscape), bottom-up (niche changes gain influence), or processes at the regime level, which lead to an integration of innovations from the niche level into the regime (Loorbach and Rotmans, 2010; Hargreaves et al., 2013; Fischer and Newig, 2016). The former change mechanism implies the importance of contextual forces in upholding dominant systems. The latter two change mechanisms imply the importance of bottom-up niche innovations, which diverge from and challenge existing regime systems. The transition management literature acknowledges the dynamic interplay between top-down forces of contextual factors and bottom-up influences of actors (Fischer and Newig, 2016; Geels, 2020), brought about by the repeated performance of normative or divergent practices (Hargreaves et al., 2013). Yet these conceptualizations do not adequately capture how everyday actions of individuals contribute to and are influenced by sociocultural forces and vice versa. This is reflected in the critical analysis of the transition management literature by Loorbach et al. (2008, p.310), who concluded that “although experiments also involve societal and institutional aspects, they are still insufficient to amount to a fundamental debate, let alone change, at the level of societal culture and structures.”

Literature on organizational change processes echoes that both organizational factors (e.g., size and structure) and individual factors (e.g., attitudes, beliefs, and sociodemographics) influence the actions of individuals and the group (Williams et al., 1989; Mullins, 1999; King and Lenox, 2000). Tudor et al. (2008), for example, suggest that the best framework for understanding change in an organizational setting incorporates individual and organizational factors as interrelated, integrated, and dynamic processes. However, for decades, organizational “change” was dominated by a discourse of “stability” (Orlikowski, 1996). In fact, most organizing discourses continue to be premised on the primacy of organizational stability (e.g., planned change models, technological imperative and punctuated equilibrium; Tsoukas and Chia, 2002). These narrow considerations of organizational change, which see it as abrupt, radical, planned and/or top-down are limiting, as change is seen as something “unusual.” Instead, scholars point to the importance of considering change as normative in processes of “organizational becoming” rather than “organizational being” (Tsoukas and Chia, 2002). Every action by an organizational member either reproduces existing organizational properties or alters them (articulated by Giddens, 1984 as social practice theory). Organizational change in this sense is inherent in everyday human actions, not inherently based on stability (Orlikowski, 1996).

Applying these notions to sustainability-related change efforts in HPBs demands an integrated perspective that equally considers individual agency and structures, and the inherent power of human actions as a driver of change. Researchers are increasingly pointing to the importance of an organization’s culture as integral in shaping the actions of organizational members (Linnenluecke et al., 2009; Salvioni et al., 2017; Adams et al., 2018; Bauer et al., 2020; Niedlich et al., 2020). Change initiatives are most likely to succeed when they are compatible with the existing (organizational) cultures; or when they are not, significant cultural transformation occurs to improve this alignment (Schein, 1985). While the “cultural” dimension appears to be a fundamental dimension of the transformation toward sustainability, it has been largely neglected. In the following, we will explore how centering cultures as a key leverage point for change can help (re-)integrate dimensions of sustainability, and the roles of individual agency and structure in change (Packalén, 2010; Dessein et al., 2015; Kagan et al., 2018).

### Cultures as the Leverage Point for Sustainability

Foremost, “culture is the living, changing dynamic of how we live our lives, individually and collectively, locally and globally, consciously and unconsciously” (Worts, 2011, p. 118). It refers to all that we mean when we talk about values and norms, rituals and traditions, symbols and language (both textual and visual), and practices. Values form the underlying base and practices, rituals and language are the experiential manifestation of those values (Hofstede et al., 1990; Dreyer et al., 2018). Fundamentally, cultures are a dynamic of human relationships (Worts, 2011). We can say that collectively, we are shaped by our cultures, even if our “cultures” never reveal themselves on a conscious level. Finding out what these concealed mechanisms are is part of intercultural communication, which arguably is extremely important for social sustainabilities (Packalén, 2010). Cultures can thus be understood as dynamic change processes; and just like change, can be considered inherent in everyday human actions (Schwartz and Davis, 1981).

There is increasing recognition of the role of cultures as a prerequisite for social change, given that they represent a central value system, guarantee social cohesion and are a mode of place and identity-making (Lehmann, 2010; Barthel-Bouchier, 2012; UNESCO, 2013). Culture is also discussed as a motor for transformation, producing “creativity,” “engagement,” and “projection” (Florida, 2005; Habitat, 2013; UNESCO, 2013; Vojnovic, 2014; James, 2015). Packalén (2010, p. 119) describes that culture, through “reflection, development, and changes in our values, forms the basis for [sustainability], but also produces new culture itself.” In this sense, culture is intertwined with other important aspects like a “sustainable way of life,” providing an alternative to a neoliberal consumer culture (UNESCO, 2013; Davies, 2015). We agree with Packalén (2010, p. 118) that the change required for ensuring truly sustainable futures “can only succeed if we consider it a necessary undertaking for the whole of society, as a great, culturally transforming, creative task, as a kind of ‘concrete utopia.’” Thus, sustainability “should be more thoroughly thought through and extended so that the cultural dimension is on par with, or rather permeates, the ecological, economic, and social dimensions like a red thread running through a thick rope, clearly visible for all to see” (Packalén, 2010, p. 119). In this vein, one could conceive of three roles for culture: culture *in*, culture *for*, and culture *as* sustainability (Dessein et al., 2015).

First, culture can have a supportive and self-promoting role (characterized as “culture in”). This expands conventional sustainability discourse by adding culture as a self-standing fourth pillar alongside separate ecological, social, and economic considerations and imperatives (Thiele, 2013). Second, a role (“culture for”), which offers culture as a more influential force that can operate beyond itself. This role moves culture into a framing, contextualizing, and mediating mode that can balance all three of the existing pillars and guide sustainable transformation between economic, social, and ecological pressures and needs (Worts, 2011). Third, a more fundamental role (“culture as”) sees culture as the necessary overall foundation and structure for achieving the aims of sustainability transformations. In all three roles, culture is recognized as the root of all human actions and an overarching concern (even a new paradigm) in sustainability. One can therefore see the debate about what sustainability really is as a discourse of cultures (Packalén, 2010), and cultures as a foundation of social justice, economic equity, and environmental protection. In the following, we discuss how cultures can serve as a means for working toward just and sustainable change.

### Cultures of Sustainability

A serious limitation in working toward sustainability goals is that they can be interpreted from different (potentially contradictory) ideological perspectives (Ben-Eli, 2018) and that understandings of sustainability are rarely explicitly articulated in change efforts (Agyeman, 2005b; Davidson and Venning, 2011). We recognize that sustainability’s diverse interpretations have emerged from social processes. Further, because sustainability (and even more so sustainable development) is a normative concept, defined in a Western cultural context, it may conflict with non-Western cultures (Meuleman, 2013). Thus, to operationalize the concept and allow for informed change efforts, especially among stakeholders with differing perspectives (Pope et al., 2017) its principles and criteria must be clearly articulated.

We perceive sustainability as a concept whose meaning emerges organically from conversations about desired futures that are informed by some understanding of the ecological, social, and economic impacts of different courses of action (see Robinson, 2004; Riemer and Schweizer-Ries, 2012). Harré (2011) has argued that if we keep looking at sustainability as a kind of problem to be solved, we will be vulnerable to arguments that suggest that any of the solutions, we propose are not good enough. Thus, it is useful to think of sustainability not as goal to work toward that is fully achievable, but rather as a compass, which will help us to keep in the right direction of a continually ongoing process of change (Harré, 2011; Thiele, 2013). Any criteria for what cultures of sustainability may be, must be developed through a collaborative process. The views of Morrison-Saunders and Therivel (2006) on public participation and the delivery of sustainable outcomes are thus instructive. The authors note that inclusion through consultation alone may not lead to socially optimal solutions. The most vocal and persuasive members of the public – often those most likely to be on committees and steering groups – may not represent the views of the wider public. Therefore, ongoing participation is integral to the process and ensures that outcomes are shaped by all stakeholders rather than *ad hoc* consultation that incorporates only a limited temporal and spatial sample of community views (Clark, 2018; Hügel and Davies, 2020). Currently, those involved in debates about sustainability are mainly politicians, activists, transition management, or other experts, but rarely ordinary citizens. Yet if the general public is to understand what sustainabilities are and if their voices are to be heard, criteria for sustainability “should be drawn from broad representation of key grass-roots, professional, technical, and social groups, including youth, women, and indigenous people – to ensure recognition of diverse and changing values” (Hardi and Zdan, 1997, p. 3).

We define COS, as characterized by *shared values, symbols, rituals*, *and practices grounded in sustainability principles* leading to individual and societal choices that promote environmental protection, social justice, and well-being, and a supportive economy (Marcus et al., 2010; Riemer et al., 2014). We find it useful to echo Worts (2011), who describes various continuously evolving capacities, at individual and collective levels, that cultures of sustainability could include, for example, capacities for participation/engagement in what is relevant, for relatedness, compassionate connection to others and to the environment, for conscious systems of knowledge, including values, for responsible action, (and) for ability to embrace change. These capacities highlight the importance of fluidity, process, and human action; fundamentally it focuses on capacities, which recognize the importance of a simultaneous focus on structure and agency (Dittmer, 2019).

To summarize, when considering sustainability in the built environment it is important to consider the complex interactions of the physical structure of HPBs with building citizens as individual agents and organizational social structures. The review of the organizational change literature further identified individual and organizational factors as interrelated, integrated, and dynamic processes. Cultures – that is, the interaction of values, practices, rituals, and symbols – are a central interlay connecting individual, organizational, and physical factors in working toward sustainability related outcomes. This highlights the need for a systems approach for fostering sustainability in this context. Likewise, cultures serve as a foundation of social justice, economic equity, and environmental protection. As such, a focus on cultures offers a much-needed alternative application of this tri-factor of sustainability, especially with respect to social justice. Human actions as a driver of needed structural changes, which in turn impact individuals’ actions, create a continuous reinforcing feedback loop. Thus, transitions to cultures of sustainability in this context need to be fostered through a bottom-up approach of engaged building citizens. This bottom-up engagement process is the second key aspect of our theory of change.

## A Theory of Change: From Design to Cultures

**Figure 1:**
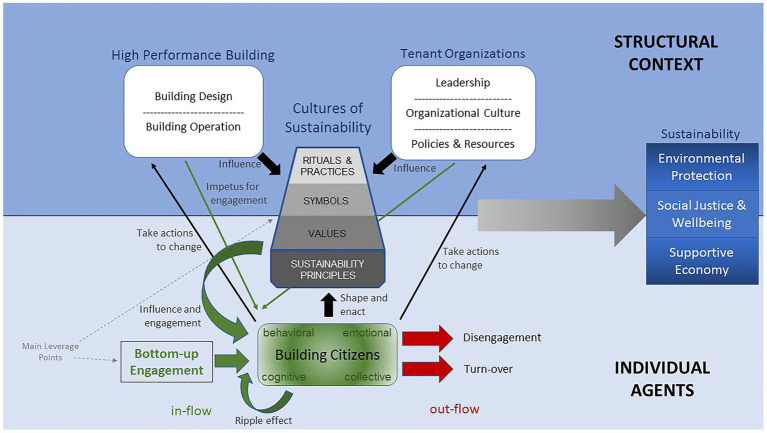
Theory of change for creating a culture of sustainability in green buildings.

As can be seen in Figure 1, the system is conceptualized as a complex interaction among structural elements and individual agents with COS at the intersection of those two layers. Engaged building citizens are the key agents and drivers of that COS by shaping and enacting values, symbols, rituals, and practices. COS, in turn, influences and engages building citizens, and as such constitute the key reinforcing feedback loop. It is within this feedback loop that we locate the opportunity for intervening in the system through bottom-up engagement and building a strong COS. The more building citizens are engaged in a COS (the in-flow), the stronger, more influential, and durable is the COS. On the other side, if engaged citizens become disengaged or leave for another office building, then the stock of engaged citizens declines (the out-flow) and the COS may weaken. The HPB and the tenant organizations in the building both serve as an impetus for citizen engagement and influence the COS (e.g., by communicating sustainability values). In the following, we will first elaborate the systems thinking that informed this model before turning to the engagement process as the key approach to intervening in this system.

### Thinking in (Building) Systems

Building design and organizational change may not seem related, but the two elements have a symbiotic relationship. Certain building features influence an individual’s actions and experience in complex ways (Coleman, 2016; Dreyer et al., 2018; Spadafore et al., 2021; Zitars et al., 2021). For example, a centrally located, open, and inviting staircase can increase the use of stairs over the elevator, while also communicating sustainability as a value to both citizens and organizations. Similarly, a café in the building that contains inviting spaces to interact with each other and features local, healthy, organic, and fair-trade items promotes community-building and again, communicates sustainable values and facilitates sustainable practices. These are just two examples of how two seemingly unrelated elements, the physical space and the decision of individuals, are connected and can ultimately lead to organizational change.

Meadows (2008, p. 2) describes a system as “a set of things – people, cells, molecules, or whatever – interconnected in such a way that they produce their own pattern of behavior over time.” This includes “adaptive, dynamic, goal seeking, self-preserving, and sometimes evolutionary behavior” (p. 13), just like a forest that is composed of a complex interplay of trees, bushes, mosses, and animals. Senge (1991) has demonstrated that within a systems context, actions that appears rational from the perspective of an individual actor can unintentionally contribute to significant problems that undermine the system as a whole. Systems thinking is a set of synergistic analytic skills used to improve and understand the system as a whole, by identifying underlying systemic structures and understanding how different system parts work together to produce specific practices and devise modifications to them in order to achieve desired goals and objectives. Once the system and its dynamics are better understood, leverage points for intervening in the system and creating transformative change can be identified (Meadows, 1999, 2008).

While the social behavior within and between different organizations in a multi-tenant office building can be viewed as a complex system itself (Dooley, 1997; Holland, 2006), the physical building adds an additional dynamic, especially when the focus is on fostering sustainability in HPBs as in our case. Porter and Cordoba (2009) identified three broad categories of systems thinking that can be applied to the sustainability debate: functionalist, interpretative, and complex adaptive. We investigate change in HPBs *via* the framework of complex adaptive systems, which are both self-organizing and learning (Dooley, 1997; Holland, 2006) and reflect a “bottom-up approach emanating from large populations of independent, interacting, and self-interested agents” (Davidson and Venning, 2011, p. 215). An essential characteristic of such systems is its emergent characteristics and nonlinearity, leading to multiple possible outcomes of dynamics. In complex adaptive systems, taking inadequate account of the inter-relationships between objectives and outcomes, can result in negative unintended consequences, such as performance gaps in HPBs (Shrubsole et al., 2014). Thus, any engagement with such a system, whether practice- or research-oriented, demands project design, measurement, and evaluation tools that are suited for such complexity (Shrubsole et al., 2019b). The systems thinking approach thereby contrasts with traditional analysis (reductionist), which studies systems by breaking them down into their separate elements. Jay Forrester of MIT and his students set the groundwork for understanding and modeling complex system dynamics within organizations (Sterman, 2000). They highlight that parts interact with each other as an interconnected set of reinforcing and balancing feedback loops. Some of the system’s impacts or outcomes develop over time and sometimes can be quite delayed and not immediately noticeable. Taking away paper towels in the public washrooms in an office building, for example, may reduce paper waste and costs in the short-term, but it can also create resentment toward sustainable initiatives when people relied on those paper towels for a variety of purposes (e.g., cleaning up a spill in their office) or if the available air dryer is not working well. As people get increasingly annoyed with this situation and talk to each other about it, the resentment builds and may interfere with future initiatives. Systems thinking provides tools to anticipate some of the unintended consequences and figure out ways to avoid them.

The system change framework developed by Foster-Fishman et al. (2007), builds upon earlier system theories by Forrester, Meadows, and Sternman and provides a useful approach to modeling the complex, dynamic, and multi-level interactions between the two major systems within HPBs: (A) The physical side (the building design and features), which defines our system boundary, and (B) The people side including the tenant organizations and the building citizens (Coleman, 2016). Key actors on the people side in this system include the employees (as the main occupants), owner, tenant management, building management, and staff, and the surrounding community interacting with the building. Fundamental systems parts related to the tenant organizations include their leadership, organizational culture, resources, and regulations/policies (Foster-Fishman et al., 2007). In this model, specific cultures are developed among building citizens interacting with each other and building features (some of which are in return influenced by citizens such as personal plants and artwork) and influenced by other system parts (e.g., policies, leadership). Over time and through various building phases (pre-occupancy, transition, and post-occupancy) these system components interact in unique ways, shaping the creation of the COS and the actions of building citizen and their experience in the building (Coleman, 2016), in turn influencing the resource use of the building as a whole as well as other dimensions of sustainability. The success of HPBs has traditionally been gauged by the how closely they meet the (mostly emissions-based) performance goals rather than illustrating how they function as part of this integrated system (Cole, 2012). As such, understanding the building as a system is crucial in creating COS, which then supports the performance and sustainability goals of HPBs.

In this paper, we primarily discuss the cultural aspect of this system dynamics model. However, it is important to consider other systems components that may be connected through reinforcing and balancing feedback loops (Sterman, 2000; Meadows, 2008). For example, inspired by a series of informational workshops and vegetarian cooking classes, the employees of an organization may develop a collective value for reducing the environmental impact of the food they consume during meetings. After advocating for a change with the organizational leadership, this shift in values may result in a new company policy of only allowing plant-based meals for official meetings. This policy, in turn, will then communicate the value underlying the policy to new people joining the company. The key to transformative change is to find leverage points in the current system that can bring about desired changes in the system (Foster-Fishman et al., 2007; Meadows, 2008). Detecting leverage points typically requires the participation and collaboration of different system actors to understand the dynamics within and across specific parts of the system. Meadows (1999) found that changing systems norms and mental models is one of the most effective leverage points for creating truly transformative system changes, which is why the focus on culture is so critical to our theory of change. Yet, it is not sufficient to just incorporate the key characteristics of systems thinking into cultural change strategies. If the goal (COS) and objectives (e.g., closing the performance gaps) are not underpinned by clearly articulated sustainability principles that are endorsed by building citizens (as discussed in the previous section), identification of impetus and engagement strategies will be unclear during cultural change initiatives.

Bottom-up engagement processes that clarify, reinforce, and support the creation of principles consistent with COS are critical to ensure evaluation and re-assessment are embedded into the change process. If these are absent, the validity or the capacity of cultural change processes to deliver COS outcomes is rendered doubtful. Thus, we will now turn our attention to participatory engagement processes necessary in fostering change toward COS within the system boundaries.

### Bottom-Up Change Through Engagement

A truism of organizational change is that senior management must fully support any transformational program (Danter et al., 2000). Wang et al. (2020) argue that reaching sustainability goals within HPBs is not possible without the participation of key internal stakeholders, as they are responsible for projects and actions, in addition to being affected by their implementation. Yet, as argued previously, top-down processes alone are insufficient for cultural change processes, so collective bottom-up efforts are required. For this to be effective, significant engagement is needed from the building citizens.

Engagement is a conscious process that is more comprehensive than behavior manipulations (Meyer and Gagnè, 2008; Shove, 2010). Engagement occurs across cognitive, emotional, behavioral, and collective dimensions; ideally all four simultaneously (Riemer et al., 2014). Engagement strategies grounded in this understanding focus on developing ongoing community and providing different options to connect cognitively, emotionally, behaviorally, collectively to sustainability over time (Macey and Schneider, 2008; Meyer and Gagnè, 2008; Riemer et al., 2014). Thus, engagement focuses on actions (e.g., language, rituals, and practices) that contribute to cultural change. As suggested by Senge (1991), people do not necessarily resist change, they just do not like being changed without their input. In this bottom-up approach, building citizens become promoting agents and not just recipients of sustainability policies and regulations. An engagement process is not about manipulating a person to do the right thing against their will, but about activating existing energy. That is, a person needs to have at least some initial openness to sustainability or related issues (engagement potential) or an external element that opens a space for action (impetus). Then they make a conscious decision to become more engaged (through a spark). What provides an impetus for one person or the other is not equal. For some, a shared kitchen is impetus to eat lunch away from the desk and begin interacting with other building citizens, while for others it is the invitation through a colleague to join them. In our model (see Figure 1), physical design features, aspects of tenant organizations (e.g., a new policy or an onboarding video), the existing COS, as well as other citizens (through a ripple effect) can serve as impetus for engagement.

Engagement processes for social change cannot be forced, they can only be fostered. They require enough individuals with an engagement potential, which in turn requires time, resources, and long-term commitment (Riemer et al., 2012). Our change framework relies on the development of supports and services needed, and a dedication of necessary resources to the bottom-up engagement program. Once citizens who have engagement potential and impetus make a conscious decision, or “spark,” we need to ensure that people can engage; that they have the time and supports required (e.g., green team, Manager of COS). Engagement is thus something that needs to be implemented actively and intentionally. An application of systems thinking further points out that certain desired actions require changes to the social-ecological system that can either enable or hinder specific further actions. For example, if sourcing local food may be challenging for employees, they could advocate for a weekly farmers’ market at their building. The search for sustainable futures “requires connecting knowledge to the capacities and capabilities to make desired changes” (May and Perry, 2006, p. 30). It is assumed that more active engagement efforts are needed initially, while over time a strong COS and a high number of engaged citizens sustain engagement through a reinforcing feedback loop. However, disengagement (e.g., because of competing demands and lack of time) and employee turn-over can negatively affect the strength of that loop, which will likely require ongoing intentional engagement efforts to counteract that decline.

Citizens also actively shape their structural environment through specific actions. In the area of environmental protection, Alisat and Riemer (2015) have defined the concept of environmental actions as ranging from low-level participatory civic action, such as informing oneself about environmental issues and participating in community events, to highly involved and political leadership actions such as organizing a protest. Engaging in these types of actions often requires specific types of competencies, which Jensen and Schnack (1997) refer to as action competencies. More recently, Dittmer et al. (2018) identified four elements of these action competencies: knowledge about the issues, reflection on knowledge and experience within the context of one’s values, visions for alternatives, and the ability to engage in collective action. Similarly, in their call for a shift in individual and collective mindsets to effectively engage in climate action, Wamsler et al. (2020) developed a competency framework of five clusters of transformative skills and qualities necessary for shifting mindsets related to climate action. These are (1) openness, self-awareness, and reflection; (2) compassion and empathy; (3) perspective-seeking and relationality; (4) agency, empowerment, and sense-making; and (5) values-based courage and engagement. Some people will have already developed these competencies, while most people have not. Creating structures and mechanisms that function as experimental safe spaces is central to supporting the development of such competencies (Wamsler et al., 2020). Our theory of change also incorporates “Assess & Adapt” as ongoing processes that serve to learn about stakeholders needs and competencies, through an understanding of their internal landscape and current COS. This identifies crucial leverage points and prioritizes time/resources based on gaps in the process. An ongoing assessment and feedback system then allows for continual improvement through; pre-occupancy and post-occupancy focus groups, annual building surveys, and interactive research projects, such as photovoice research, among others.

Engagement is crucial not only in terms of the delivery of the change strategy but also in the very framing of the goals/objectives of the COS. Engagement processes are not about presenting goals and ready solutions to stakeholders; as discussed above, the simple inclusion of stakeholders is not sufficient in ensuring that sustainability goals are met. *Co-creation* of goals and strategies, requires design thinking and well facilitated group processes (see Geobey, 2021). The assumption is, that over time, after being reinforced by their surroundings (both physical and social), building citizens who were slightly engaged originally will be part of a ripple effect. Research shows that we are heavily influenced by our immediate social group and diffusion of innovation and social change often starts with a few individuals (the innovators and early adopters) but then ripples to others within their social group (Rogers, 2003). Over time, this can result in cultural changes within an organization or community, which then, in turn, influences further engagement. As such, culture is a powerful means to elicit engagement. Mintzberg and Westley (1992) suggests that organizational culture is equivalent to the soul that binds people and organizations together and it guides organizational members’ believing and thinking, perceiving and feeling, ultimately directing their behavior (Smircich, 1983; Schein, 1985). Engagement and cultural change are mutually reinforcing mechanisms, which are both fluid without a determined end state; culture can be arguably experienced and expressed cognitively, emotionally, behaviorally, and collectively; and engagement across these dimensions lead to actions that change cultures.

## From Theory to Action

With the core elements of the theory of change and their relationships laid out and justified, implementation and translation into action follow. Based on the theoretical consideration above, our team developed a manual (“Momentum for Change: A Culture of Sustainability engagement manual”) that served as a general guideline for key change agents in developing an applied collaborative COS engagement strategy (Riemer et al., 2018).^4^ In this translation from theory to proposed action, it is important to consider that the specific actions cannot be pre-determined or prescribed as that would go against the co-creative bottom-up approach and would ignore the specific cultural and organizational contexts. Rather, it is important to present a set of principles that can be applied across different contexts and interpreted collaboratively by local actors. In our case, we derived five core principles for the development of the strategy: systems-oriented, long-term developmental, strategic, comprehensive, and participatory (see Table 1 for an overview).

**Table 1:** Core principles derived from the theory of change.

Element	Intention
Systems-oriented	Rather than focusing on only changing a single element of a social system the approach will identify key leverage points in the system for transformative and durable impact
Long-term developmental	The engagement processes are built on relationships between people and mobilizing them in experimentation. Through both successes and failures these experiments create opportunities to deepen bonds of trust and integrate systematic learning into the process.
Strategic	There is a long-term strategy with a clear vision and general purpose, long-term and intermediate goals, specific objectives, general strategies, and specific actions
Comprehensive	The engagement strategy is multi-dimensional, targeting cognitive (thinking), emotional (feeling), behavioral (doing), and collective (being) dimensions, and also works across multiple scales from the individual, to the organizational, to the entire site with the ultimate goal of having impact on communities beyond evolv1. This requires multiple interventions rather than attempting to find a single solution to rally all stakeholders to support.
Participatory	Employees, managers, and other building citizens will use their own information, experiences, and capacities to develop “local theories” about the causes of problems and how to solve them. Through a cyclical problem-solving process, the people in the building will co-design and implement a series of solutions and learn from their results.

Source: Riemer et al., 2018.

First, our theory of change foregrounds understanding HPBs as complex and dynamic systems with three interconnected components: the physical building and the social system, which includes the tenant organizations and the building citizens. A key focus is on the emerging COS as a major mechanism for transformations that will foster enduring sustainability that permeates each tenant organization. Second, this type of transformation necessitates a critical mass of engaged building citizens (the stock) who are collectively changing shared values, social practices, rituals, and symbols/language. This is an ongoing, relational, dynamic, multi-year process that we believe can only be fostered but not directed. Third, this type of approach requires a long-term strategy with interconnected strategic actions that build upon each other. For example, it may be important to first develop relationships and community among building citizens (i.e., occupants and building managers and staff) before larger collective goals can be pursued together. Engagement of building citizens is the key driver of cultural change in this approach. Fostering this level of engagement needs to be a multi-level and multi-dimensional effort across an array of interventions that target cognitive, emotional, behavioral, and collective aspects of engaging with sustainability. Finally, the focus on bottom-up approaches to fostering collective engagement and co-creative processes by the building citizens is captured in the participatory principle.

In the application of these core principles, we developed a multi-year strategic plan. This included the use of participatory design workshops to determine what sustainability means to us, forming a building COS committee, hiring a COS manager to foster bottom-up engagement, creating opportunities to develop community, and increasing the capacity for collective actions, among other specific strategies derived from the general principles. This plan also included strategies to leverage the intentional interior design elements of the building, and created opportunities for an increased awareness of the physical space through building tours. Interest and awareness of the research and building was fostered through informational material provided for new employee onboarding. We also worked with tenant management to communicate sustainability as an organizational value using the building as the impetus for that.

## Potential Challenges and Solutions

Bickman (1987, p.5) defined program theory as “a plausible and sensible model of how a program is supposed to work.” Often, however, what sounds plausible and sensible in theory will be challenged once you try to implement it in practice in specific context with all of the messiness and competing demands that exist in those real-world contexts. There are a few specific challenges, we were anticipating in implementing such a comprehensive and long-term approach as is represented by our theory of change.

1. Changing cultures takes a long time. There is danger of losing momentum if there are no quick wins. Competing demands can also lead to disengagement. Another challenge can be losing key champions who have a lot of weight in carrying the change process. Turnover is a common challenge in organizational change efforts. Thus, thinking about redundancy early is important.
2. The sponsors of change initiatives at the organizational leadership level may prefer quick-fix solutions that focus on the individual over investment in a comprehensive long-term system-change strategy. While quick-fix solution are less likely to create meaningful and long-lasting change, they may satisfy the need to include something into the corporate sustainability report or to feature on the company’s website. Therefore, a good long-term strategy may include some initial actions or programs that can lead to quick wins to ensure the continuous buy-in of the organizational leadership.
3. Companies have realized that creating positive organizational cultures is key to attracting and keeping the modern workforce, especially younger mobile employees (Fernandes, 2018). Thus, creating a COS may compete with other efforts of creating organizational cultures unique to each tenant organization within the building. For that reason, it is recommended to identify such efforts and integrate and align the COS strategy with these other efforts.
4. While a lot of individual behavior change strategies have been intentionally designed to not even require the target individuals to be consciously aware of the processes, engagement on the other hand takes conscious effort and time that many may not have and will not be given by the supervisors. This may especially be an issue in team-oriented and project-based work that has replaced the more traditional 9–5 types of jobs (Matthews et al., 2018). The nature of this type of work makes it harder for individual workers to justify their engagement in things other than the project-oriented work. Asking organizations to provide regular designated “sustainability hours” that can be used to work on individual or collective sustainability actions may be a way to address this.
5. Fostering cultural changes operating at a systems-level are resource intensive. Unless sustainability is seen as a key organizational priority, this may not be an investment organizations’ feel like they can make. Thus, it is important to ensure the level of organizational commitment before engaging in the change process.
6. Finally, multi-tenant buildings pose unique challenges because one has to deal with different organizational cultures, structures, and procedures. It also requires more upfront investment in relationship-building and developing community among employees from different organizations.

## Conclusion

Systems theory suggests that the most impactful and long-lasting changes in social systems target system elements with high reach or influence (Murphy and Jones, 2020). Applied systems thinkers often use the iceberg model to illustrate these highly influential system layers for lasting transformative change. Accordingly, the behavioral level (which is the top layer of the iceberg that can be seen above the water surface) is the least transformative leverage point, while the deepest level (under the water surface), that is values, mental models, and cultural beliefs, is the most impactful one (Senge, 1991; Meadows, 1999; Murphy and Jones, 2020). Of course, this level is also the most complex and difficult to influence, which may be the reason why there are less applied theories in psychology that are targeted at this level, while there are plenty of theories focused on individual behavior. With this paper, we hope to contribute to an exploration and discussion within psychology of how we may develop systematic approaches to intervening at these deeper levels and offer this unique project as a promising starting point for this conversation.

The original motivation for the theory of change presented in this manuscript was the request by the leadership team to create a behavior change strategy to avoid the before mentioned performance gaps often observed for HPBs. Our team determined that creating a strategy that would change multiple actions simultaneously and maintain these changes over time, as building citizens transition in and out of the building, can only be accomplished by going deeper – below the water surface of the iceberg – by creating cultures of sustainability through building citizen engagement. Beyond just sustainable building design, construction, and operation, cultural change initiatives undertaken with meaningful engagement have the potential to result in a more robust prototype than any single case study building. Leading not only to carbon reductions within HPBs, but also movement toward economic equity, environmental protection, and social justice (including health and well-being).

In many existing psychological theories, cultural factors are recognized as crucial influencing factors, for example, values are prominent in most individual-level behavior change strategies (e.g., value-belief-norm theory; Stern et al., 1999), yet they rarely are considered *the* focal element for transformative change. Yet, transformative change toward sustainability demands that scientists, intellectuals, and other professionals recognize the limits of current theories of change. Sustainability implies a change of fundamental cultural epistemologies and hence a fundamental change in our scientific models and approaches (Reason, 2002; Sterling, 2004). Recognizing the public role of science, many scholars further problematize the linear, instrumental perspective between institutions of higher education, research and learning and the solutions of social and political problems, such as sustainability challenges (Van Poeck and Vandenabeele, 2014). Working from within the discipline of community psychology, we also embrace the centrality of issues of justice in cultural transformations. If sustainability is to become a process with the power to transform, “justice and equity issues need to be incorporated into its very core” (Agyeman, 2008, p. 752); only then can we truly realize the potentials of HPBs. Working within current neoliberal market structures, we recognize the limitations of this economic model in supporting transformational change that is not linked to increasing growth-oriented sustainable development. Engagement of employees is only possible, if organizations encourage employees to allocate their workhours toward shared building and community-level goals. Changing cultures is extremely difficult and requires long-term commitment that many organizations may not be prepared to make.

Buildings can be more than physical spaces, we occupy, they can foster a sense of shared identity, the feeling of recognition and of belonging to a specific place that improves quality of life. When they are designed as a collective construct, a feeling of co-responsibility informs our efforts. They can then provide reference points to which people can relate and connect – a culture. We hope this paper provides organizational change agents with a framework they can use in the development of their comprehensive change strategies. However, cultural change requires more than a cookie-cutter approach, or recipe that one can simply follow, but rather general principles that require an understanding of the underlying theory of change, which is why we elaborate on ours here. This 5-year study also aims to address the significant gap in the literature regarding the empirical evaluation of such comprehensive co-creative approaches. We also hope that it gives researchers a starting point if they are looking for approaches that go beyond incremental behavior change and involve co-creation toward more just and sustainable societies.

## Data Availability Statement

The original contributions presented in the study are included in the article/supplementary material, further inquiries can be directed to the corresponding author.

## Author Contributions

All authors contributed to the development of the theoretical model and intellectual conceptualization of this paper. Author contribution reflects the relative contribution of each author to iterations of the manuscript. Sharing first authorship, BD and MR contributed equally to the writing and intellectual conceptualization of the current version of this paper, with significant contributions from BS. DF, AT, JM, SG, and AD all wrote sections of the original manuscript, with JM, SW, and AD providing detailed feedback throughout the iterations of the manuscript, which this final version draws from theoretically and intellectually. All authors contributed to the article and approved the submitted version.

### Conflict of Interest

The authors declare that the research was conducted in the absence of any commercial or financial relationships that could be construed as a potential conflict of interest.

## Footnotes

^1^Cultures of sustainability are referred to in the plural to recognize that there are many different cultures of sustainability, and not one distinct “culture” that can be defined as such (see also Kagan, 2010 for a more thorough discussion).

^2^We would like to acknowledge the excellent suggestions by the two reviewers that led to further integration of more recent literatures.

^3^Most physical design features of HPBs (and other buildings) are still decided in a top-down process with little engagement. Yet, engagement should not start with the transition into the new or retrofitted building. There is growing literature that points out that many performance gaps in HPBs are related to a lack of engagement processes early in the design phase of the building. As Reed (2007) describes it, this process of integrated design shifts the role of the architect/planner/designer away from the expert holding all the knowledge to that of a facilitator of a process of revealing. Integrative design can thus create more bottom-up physical features.

^4^This unpublished manual is available from the second author upon request.
